# Identification of malaria hot spots for focused intervention in tribal state of India: a GIS based approach

**DOI:** 10.1186/1476-072X-8-30

**Published:** 2009-05-20

**Authors:** Aruna Srivastava, BN Nagpal, PL Joshi, JC Paliwal, AP Dash

**Affiliations:** 1 National Institute of Malaria Research, 20 Madhuban, Vikas Marg, Delhi – 110091, India; 2 National Vector Borne Disease Control Programme, 22 Sham Nath Marg, Delhi 110054, India; 3 State Health Department, Madhya Pradesh, India; 4 National Institute of Malaria Research, 22 Sham Nath Marg, Delhi- 110054, India

## Abstract

**Background:**

In India, presently malaria shows a declining trend whereas *Plasmodium falciparum (Pf) *cases show an up trend. In central India, specifically, Madhya Pradesh (M.P.) a forested and tribal area, control of malaria is logistically difficult and outbreaks are frequently recorded, reasons for this being inadequate surveillance, poor reporting, a time lag in reporting to decision makers and a lack of geo referenced information to pin point the trouble spots for a timely preventive action.

**Results:**

An information management system based on Geographic Information System (GIS) using district and block wise malaria data, has been constructed for Madhya Pradesh for quick retrieval of info and dynamic generation of maps to highlight hot spots of malaria for formulating prompt and focused malaria control strategy. Out of total 48 districts consisting of 313 blocks, based on certain criteria GIS identified 58 blocks falling in 25 districts as Hot Spots. Malaria flares up from these pockets whenever favourable conditions for transmission occurs. It was suggested to National Vector Borne Disease Control Programme (NVBDCP) that focused malaria control in these hot pockets may be taken up on priority during the year 2007, it was implemented by State Health Authorities, M.P. under the directive of NVBDCP. Implementation of control measures were evaluated by NVBDCP.

**Conclusion:**

GIS mapping would make it easy to update information instantly and to identify the trouble spots at the village level within the district which is the lowest unit equipped with computer facilities and the information can reach instantly to state and the policy makers to formulate focused and cost effective malaria control strategy. This is the first time when GIS has been used in national control programme for tribal malaria.

## Background

In India, the incidence of 6.47 million malaria cases in 1976 were reduced to around 2.5 to 3 million cases annually until 1996. After 1996, there was a declining trend in malaria incidence and in 2003, about 1.87 million cases of malaria including 0.86 million *Plasmodium falciparum *cases and 1006 deaths were reported from the country. Since then the reported cases were < 2 million and an increase in *Pf *cases was observed [1]. In India, tribal population (8%) contributes about 30% of total malaria cases in the country with > 60% of *P. falciparum*, and about 50% of the deaths due to malaria. Largest concentration of tribal population about 15.4 million, is found in Madhya Pradesh (M.P.), which is about 20.3% of M.P.'s total population [2,3]. Due to the different linguistic, cultural and geographical environment, and its peculiar complications, the tribal world of Madhya Pradesh has been largely cut-off from the mainstream of development and is a cause for concern for the Government [3].

In MP, the number of deaths due to malaria is on the increase, mainly due to the frequent outbreaks causing high morbidity and mortality e.g. an outbreak in Sidhi district in M.P. in 2005 and 2006 caused 25 deaths annually due to *P. falciparum*. The actual death toll was estimated to be much higher as these deaths were recorded only in the district hospital, where many patients could not reach because of inaccessibility and lack of a transport system. In M.P. the presence of two highly efficient vectors namely, *Anopheles culicifacies *and *An. fluviatilis*, thinly populated area, poorly clothed ethnic tribe scattered in fields and forest, high degree of mobility, poor health infrastructure and increasing drug resistance among *P. falciparum *are some of the factors that maintain malaria as an important public health problem. Efforts are now being made to control malaria by integrating the existing tools, such as indoor residual spraying with synthetic pyrethroid, provision of insecticide-treated bed nets for high-risk groups, rapid diagnostic tests for on-the-spot diagnosis, and prompt and effective treatment. To monitor the programme and achieve effective control, a rigorous assessment of the geographical distribution of the disease is needed [4]. Geographic Information Science (GIS) owing to its inherent ability to manage both spatial and non-spatial information provides an excellent framework for disease management [5]. Therefore, to monitor the programme and achieve effective malaria control, three institutions namely, National Institute of Malaria Research (NIMR), National Vector Borne Disease Control Programme (NVBDCP) and State Health Authority, Madhya Pradesh, initiated a joint venture using Geographic Information System (GIS). The objectives were i) to assist in planning and implementation of situation specific control strategy ii) monitoring and evaluation of control measures. This is the first time in India when GIS has been used as a decision making tool for formulating control strategy for tribal malaria at the block level.

## Methodology

### GIS Based System

**Figure 1:**
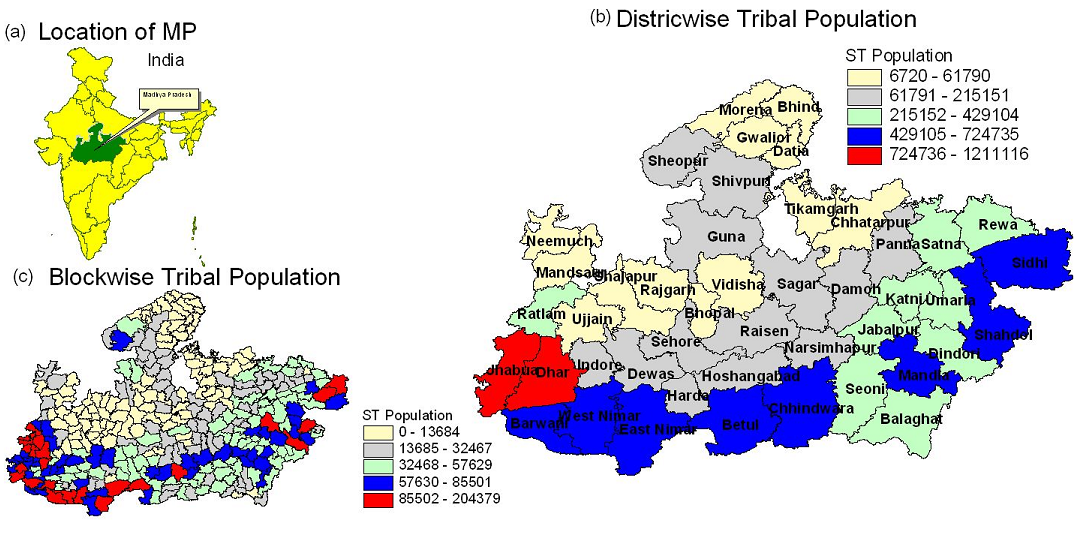
**a) Location of Madhya Pradesh in India**. b) Tribal dominated Districts in M.P. c) Tribal dominated blocks in M.P.

Madhya Pradesh is situated at lat/long of 23° 30' N latitude and 80° 00' E. There are 48 districts consisting of 313 blocks. It is rural agricultural state with 31% forest covers marked by severe poverty, under development and largest concentration of tribal population [2]. Location of M.P. in India and tribal dominated districts/blocks are shown in Fig. 1a, b and Fig. 1c.

**Figure 2:**
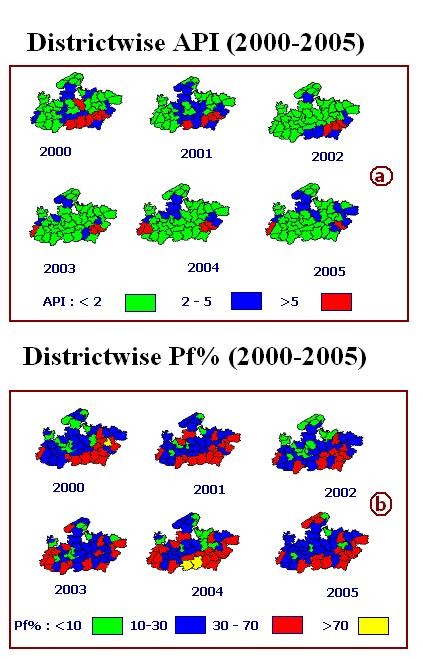
**Spatio temporal evolution of malaria in 2000 – 2005 in districts of Madhya Pradesh**. a) District wise Annual Parasite Incidence: number of positive cases per thousand population per year (2000 – 2005). b) District wise *Pf *per Cent: Per cent *Pf *cases out of total positive cases.

**Figure 3:**
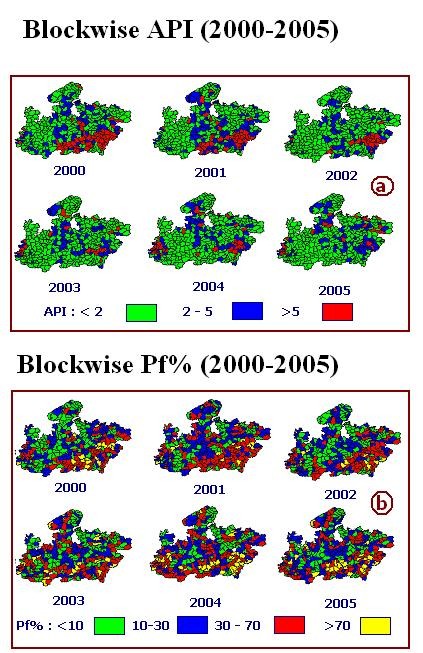
**Spatio temporal evolution of malaria in 2000 – 2005 in blocks of Madhya Pradesh**. a) Block wise Annual Parasite Incidence: number of positive cases per thousand population per year (2000 – 2005). b) Block wise *Pf *per Cent: Per cent *Pf *cases out of total positive cases.

District and block wise geo referenced maps of Madhya Pradesh were prepared using Census Administrative Atlas for overlaying and integration of thematic layers [5]. Census 2001 data such as, population, tribe population etc. were attached to the maps [6]. District and block wise malaria data was procured through State Health Department. The data, both for districts and blocks, consist of population, blood slide collected, examined, number of positive cases for both the Plasmodium species namely, *Plasmodium vivax (Pv) *and *Plasmodium falciparum (Pf)*, etc. for six years i.e. from 2000 to 2005. The data was attached to the districts and block PHC polygons on the maps. Parasitological indices such as Annual Parasite Incidence i.e. number of blood slides positive per 1000 population in a year, *falciparum *proportion i.e. per cent positive slides for *P. falciparum *(*Pf*%) was estimated within the GIS database. For district and blocks thematic maps of annual parasite incidence and *Pf*% for each of the years 2000 – 2005 were prepared (Fig 2a, b, Fig. 3a and Fig. 3b).

Since *P. falciparum *is responsible for mortality due to malaria and also is indicative of fresh transmission, Pf% cases were considered for identifying the hot spots. The following conditions were laid down for blocks/districts to qualify as hot spot. Blocks/districts satisfying one of the following conditions during 2000 – 2005, were identified, extracted and integrated within GIS frame work to map malaria hot spots.

### Conditions for blocks/districts qualifying as Hot Spots

1. 100% *Pf *in any year in (2000 – 2005)

2. Consistently >30% Pf

3. *Pf *> 70% in 2005

For the above process following steps were followed.

**Figure 4:**
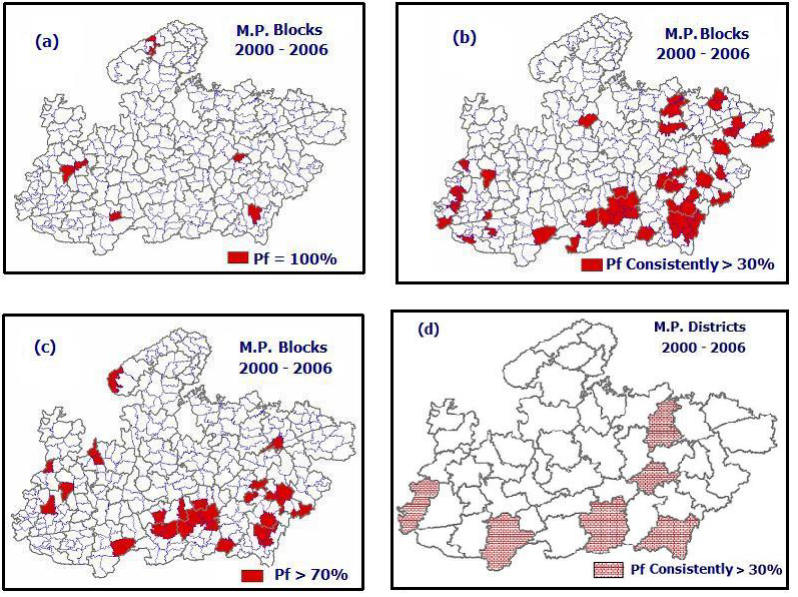
**Blocks satisfying conditions laid down for classifying as hot spot**. a): Blocks showing 100% *pf *cases during 2000 – 2005. b): Blocks exhibiting consistently >30% *pf *during 2000 – 2005. c): Blocks reporting >70% *pf *in 2005. d): Districts exhibiting consistently >30% *pf *during 2000 – 2005.



a) Blocks with 100% Plasmodium *falciparum *cases were extracted from Fig. 3b for each year (2000 – 2005) and separate layers L_1 _to L_6 _were formed. These six layers were integrated using Boolean operator ''Union' to get Layer L_7 _i.e.



where B_100 _are the Blocks having 100% *Pf *cases belonging to one or more Layers L_1 _to L_6 _(Fig. 4a).

b) Blocks with >30% Plasmodium *falciparum *cases were extracted from Fig. 3b for each year (2000 – 2005) and separate layers L_8 _to L_13 _were formed. These six layers were integrated using Boolean operator 'Intersection' to get Layer L_14 _i.e.



where B_30 _stands for blocks having > 30% *Pf *belonging to each Layer L_8 _to L_13_



(Fig. 4b).

c) Blocks having >70% *Pf *in 2005 were extracted from Fig. 3b to constitute Layer L_15 _(Fig. 4c).

Similarly using district wise epidemiological data for the years 2000 – 2005 i.e. Fig. 2b, preparation of map layers showing districts satisfying above three conditions was attempted. Since no district satisfied conditions 1 and 3, only six map layers consisting of districts having >30% *P. falciparum *cases were prepared and integrated (Fig. 4d).

The resultant map Layer L_16 _exhibiting hot spots was obtained by integrating Layers L_7 _(Fig. 4a), L_14 _(Fig. 4b) and L_15 _(Fig. 4c) using Boolean operator 'Union', i.e.



Thus, B_*o *_are the blocks in Layer L_16 _depicting hot spots in M.P.



**Figure 5:**
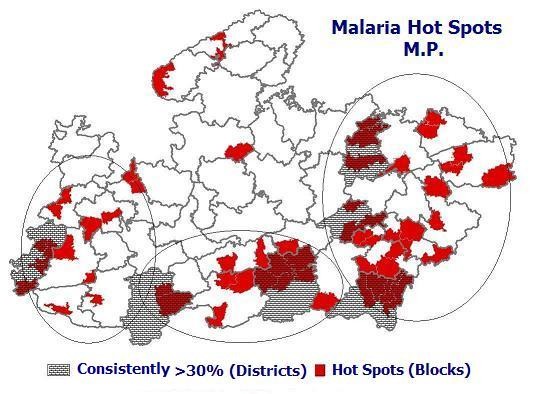
**Malaria hot pocket requiring focused intervention overlaid on problem districts in M.P. depicting geographic location for planning of logistics and schedule for intervention**.

Finally, district map in Fig. 4d was overlaid on integrated map layer L_16 _(Fig. 5) to get the final hot spot map.

## Results and Discussion

In India, under rural malaria control, during the year 'n', malaria control strategy for the coming year i.e. (n+1) is planned based on annual parasite incidence and *Pf*% of previous year (n-1), because data compilation for the n^th ^year takes about six months after the completion of the year. Therefore, as this study was conducted in 2006, for planning of control strategy for the 2007, data of API and *Pf*% corresponding to year 2005 was utilized.

Madhya Pradesh (M.P.) is located at the centre of the country and tribal populated districts/blocks mostly located on southeastern, southern and southwestern parts of M.P. (Fig. 1a, b and Fig. 1c). Fig. 2a, b, Fig. 3a and Fig. 3b depict district and block wise spatio temporal distribution of malaria from 2000 – 2005 in M. P. In general, it reveals high malaria mostly from the districts/blocks situated on southeast/southwest of Madhya Pradesh. Fig. 2a revealed that number of districts having API >5 decreased from 6 in 2002 to 2 in 2005. Eleven districts reported API in the range of 2 – 5 in 2000 which reduced to 9 in 2005. On the other hand, number of districts having >10% *Pf *in 2000 were 36 and increased to 40 in 2005, and districts having 30 – 70% *Pf *were increased from 11 in 2000 to 15 in 2005(Fig. 2b). Similar scenario is being observed at the block level. Number of blocks in the API range of 2–5 reduced from 70 in 2000 to 62 in 2005 whereas blocks with >5 API reduced from 54 in 2000 to 24 in 2005 (Fig. 3a). In regards to *Pf*%, number of blocks in lower range i.e. < 10% *Pf*% has decreased from 111 in 2000 to 82 in 2005, whereas in higher range i.e. *Pf*% in the range 10–30, 30 – 70 and > 70% have increased from 105 in 2000 to 111 in 2005, 74 in 2000 to 91 in 2005 and 20 in 2000 to 26 in 2005 respectively (Fig. 3b).

Fig 4a reveals that six blocks in five districts reported 100% *Pf *cases during 2000 to 2005. Initially, in years 2000 and 2001 no block reported 100% *Pf*, two blocks each in the year 2002 and 2003 and one block each in the years 2004 and 2005 reported 100% *Pf *cases.

Fig. 4b shows that 44 blocks falling in 20 districts consistently reported >30% *Pf *cases and Fig. 4c exhibits that 26 blocks falling in 16 districts reported >70% *Pf *cases.

At district level there is no report of 100% *Pf *during 2000 to 2005, or *Pf *>70% in 2005, however, 6 districts showed consistently >30% *Pf *incidence during 2000 – 2005 (Fig. 4d).

Fig. 5 shows integrated layers of blocks satisfying one of the three conditions (4a, b, c) and districts consistently having >30% *Pf*% (Fig. 4d) depicting the hot spots of malaria. It exhibits 58 blocks as hot spots falling in 25 districts, malaria is flared up from these blocks whenever favourable ecological conditions prevails and further defuses. Out of these 58 blocks in 9 blocks namely, Jobat, distt. Jhabua; Shahpur and Bhimpur distt Betul; Kundam, distt. Jabalpur; Bijadandi, Ghughari and Narayanganj, distt. Mandla; Amerpur and Dindori, distt. Dindorie; Jaisinghnagar, distt. Shahdol; Pawai, distt. Panna; Maihar, distt. Satna and Sidhi, distt. Sidhi outbreaks have already been reported (Neeru Singh, Personal communication).

In Fig. 1b and Fig. 1c, blocks/districts have been stratified in 5 categories depending on concentration of tribal population. The stratification was done on the basis of 'natural breaks' within GIS system in the total range of tribal population. Out of 58 hot spot blocks, 26 i.e. about 45% blocks are tribal dominated falling in two higher strata in Fig. 1c. In other blocks high risk might have been posed by migration due to several multipurpose projects resulting in the movement of people from one area to another in the tribal belt of Madhya Pradesh (MP) [7]. GIS mapping also shows the geographic location of hot pockets within districts/state to plan logistics and survey schedule in cost effective manner. It was suggested to NVBDCP that focused control in malaria hot pockets may be taken up on priority basis during the year 2007, and subsequently, it was implemented by the State Health Authorities, M.P. under the directive of NVBDCP. Implementation of control measures were evaluated by NVBDCP. Annual parasitological data for the year 2007 compiled at NVBDCP showed that 96042 malaria cases in 2006 reduced to 90829 in 2007, however *Pf% *increased [4]. In view of the utility of GIS based information management system, NVBDCP requested NIMR to carry out village wise GIS mapping for 65 high risk district in India, including 5 districts of M.P., to highlight high risk villages so to say hot spots at village level for decision support in formulation of focused malaria control strategy for the year 2009. About 50% of the work has already completed and progress is good.

District is the lowest administrative unit where computer facilities are available and is the nodal agency to forward the compiled data through State to the Directorate of NVBDCP for formulating situation specific control strategy. With the Global Fund assistance NVBDCP 's is developing a National Anti-malaria Management Information System (NIMMIS) for the improvement of supervision and monitoring the grass root level activities, The software has already been tested and full deployment of requisite hardware, software is undergoing. Application administrators, Nodal Officers, other personnel till district level (585 districts in 34 states) have been identified and trained. The data received from PHCs would be computerized at district level. It is envisaged that reporting for monitoring and evaluation would be through web based MIS [8]. NIMR is fully equipped with GIS facilities and expertise, it is proposed that two persons each from District, State and NVBDCP would be trained on GIS techniques. District headquarters would be equipped with GIS facilities and put the data on GIS platform to prepare and update maps at district headquarters. Electronically, maps would be sent to state for further transmission to Directorate of NVBDCP. The GIS system would eventually be merged with NIMMIS web-based system for global dissemination and sharing of information.

## Conclusion

GIS can dynamically map malaria hot spots it also point outs the geographic locations of hot pockets within to carry out accelerated focused malaria control by State Health Authorities, M.P. Also NVBDCP can plan logistics and survey schedule in cost effective manner. The mapping unit village, block, PHC etc and the conditions for selection of hot spots can be changed depending upon the requirement and malaria situation. The main advantage of the GIS platform is fast data updating, as soon as data is entered revised maps are ready highlighting the trouble spots, which is not feasible in the current manual system. Electronic transfer of data is much faster then postal communication. Web hosting can give a new perception to malaria data management, global information dissemination and sharing. Another advantage is that once infrastructure is ready, it is easy to convert it to surveillance system for any other disease viz. filaria, dengue, DHF. GIS-based information system ensures that if a localized spurt of disease cases occurs, it is highlighted.

## Competing interests

The authors declare that they have no competing interests.

## Authors' contributions

All authors designed the overall architecture of the system. AS was responsible for GIS database, map preparation, analysis and drafted the manuscript.

BNN collected the field data and other related information and worked for progress of the project. APD and PLJ were responsible for transfer of technology from NIMR to NVBDCP and from NVBDCP to the national programme respectively, also responsible for programme implementation evaluation. JCP was responsible for the implementation of focused interventions in the state. All authors read and approved the manuscript.
